# Prognostic impact of the geriatric nutritional risk index on waitlist mortality in adult patients listed for lung transplantation from donation after brain death

**DOI:** 10.1016/j.jhlto.2025.100424

**Published:** 2025-12-05

**Authors:** Gouji Toyokawa, Miho Yamaguchi, Takafumi Yamaya, Takaki Aakamine, Mitsuaki Kawashima, Chihiro Konoeda, Mototsugu Shimokawa, Masaaki Sato

**Affiliations:** a Department of Thoracic Surgery, The University of Tokyo Hospital, Tokyo, Japan; b Department of Surgery and Science, Graduate School of Medical Sciences, Kyushu University, Fukuoka, Japan; c Department of Biostatistics, Graduate School of Medicine, Yamaguchi University, Yamaguchi, Japan

**Keywords:** Lung transplantation, Geriatric nutritional risk index, Waitlist mortality

## Abstract

**Background:**

The geriatric nutritional risk index (GNRI) was developed to assess nutritional status of elderly patients, and is a recognized prognostic factor in various diseases. However, its role in patients awaiting lung transplantation (LT) from donation after brain death (DBD) remains unclear.

**Methods:**

We retrospectively analyzed adult patients registered for LT from DBD between January 2014 and July 2023. GNRI was calculated using the formula: (1.489 x serum albumin [g/dL]) + (41.7 x [current body weight / ideal body weight]). Its cut-off value was determined by receiver operating characteristic curve analysis for waitlist mortality. The primary objective was to clarify the association between GNRI and waitlist mortality.

**Results:**

The study included 354 patients; 147 (41.5%) survived to receive LT, while 105 (29.7%) died on the waiting list. The GNRI cut-off value was 93.84, with an area under the curve of 0.623. The low GNRI group (n = 122 [34.5%]) had significantly lower body mass index (*P* < 0.001), shorter 6-minute walk distance (*P* = 0.002), and higher need for oxygen supplementation (*P* = 0.015) compared to the high GNRI group (n = 232 [65.5%]). Multivariate analysis identified GNRI (*P* = 0.024), age (*P* = 0.047), sex (*P* = 0.013), 6-minute walk distance (*P* < 0.001), forced vital capacity (*P* = 0.023), and disease category (*P* = 0.036), as significant independent prognostic factors for survival.

**Conclusions:**

GNRI is a significant prognostic indicator for survival in patients awaiting LT from DBD, and may aid in pre-transplant evaluation.

## Introduction

Lung transplantation (LT) is a life-saving treatment option for patients with progressive end-stage respiratory diseases that are refractory to optimal medical therapies.^1^ In Western countries, the waiting period for LT from donation after brain death (DBD) is relatively short due to the large number of donors and allocation systems, such as the Lung Composite Allocation Score (CAS). In contrast, in Japan, the limited number of donors and the principle of a ‘first come first served’ system for LT result in a longer waiting time of approximately 700 days and a higher mortality rate of 33.1% compared with Western countries.^2^ As malnutrition and frailty are important indicators of waitlist mortality and post-transplant survival in lung transplant candidates, it is essential to manage the factors that adversely impact the survival of patients during the waiting period for LT from DBD.^3^, ^4^

The geriatric nutritional risk index (GNRI) is a simple composite score calculated from the serum albumin, patient body weight, and ideal body weight that was originally developed to assess the nutritional status of elderly patients.^5^ In addition to its role in predicting the risks of morbidity and mortality in hospitalized elderly patients, the GNRI has also been recognized as a prognostic indicator in various diseases, including cancer.^6^, ^7^ Furthermore, the GNRI has been reported to be associated with waitlist mortality in patients registered for heart transplantation and with mortality following heart transplantation.^8^, ^9^ In the study by Yoo et al., patients younger than 65 years old were included, and GNRI at the time of heart transplantation listing was independently associated with subsequent mortality.^8^ This suggests the potential usefulness of GNRI in a younger population, in contrast to the original study, which included only patients aged 65 years or older.^5^ However, the significance of the GNRI in the field of LT has yet to be clarified.

In the present study, we examined the association between the GNRI and patient characteristics in adult patients awaiting LT from DBD. Additionally, we conducted a multivariate analysis to determine whether the GNRI was an independent prognostic factor in this population.

## Methods

### Study cohort

Between January 2014 and July 2023, 398 patients were registered for LT from DBD at The University of Tokyo Hospital. Nine patients who received living donor LT were excluded from this study due to the short waiting period before living donor LT. We also excluded 17 pediatric patients younger than 18 years, and 18 patients with missing data for variables such as 6-minute walk distance and serum albumin level. Consequently, we included 354 adult patients and retrospectively obtained their data at the time of listing for LT from DBD in the Japan Organ Transplant Network. Data regarding LT from DBD, waiting time, and mortality were collected until the cut-off date in July 2024. The primary objective of this study was to clarify the association between the GNRI and waitlist mortality.

### Validation cohort

Between August 2023 and July 2024, 80 patients were registered for LT from DBD at our institution. Data on waitlist mortality were collected until the cut-off date in July 2025. This cohort was used to validate the results obtained from the study cohort.

### Cohort who underwent LT

Postoperative outcomes, including in-hospital death, length of intensive care unit (ICU) stay, length of mechanical ventilation, and length of in-hospital stay, in 147 patients who underwent LT in the study cohort.

### Calculation of the GNRI

Blood tests, including serum albumin, were conducted when patients were admitted to our hospital to evaluate their suitability to be listed for LT. In accordance with a previous report, the GNRI was calculated using the following formula: (1.489 x serum albumin [g/dL]) + (41.7 x [current body weight / ideal body weight]).^5^ The ideal body weight was calculated using the following formula: body height x body height x 22. The GNRI was calculated at the time of listing for LT.

### Statistical analysis

Categorical variables were summarized as numbers and percentages, while continuous variables were presented as mean or median values depending on their distribution. Overall survival (OS) was calculated from the date of listing in the Japan Organ Transplant Network for LT from DBD to the date of death from any cause. Patients who underwent LT were censored at the time of the operation. The associations between the GNRI and continuous data were analyzed using the Student’s t-test for normally distributed data, and the Mann-Whitney or Kruskal-Wallis test for non-normally distributed data. The chi-squared test was used to analyze the associations between the GNRI and categorical variables. Cut-off values of continuous variable factors, such as age, body mass index (BMI), pulmonary function, 6-minute walk distance, and GNRI, were determined based on a receiver operating characteristic (ROC) curve for overall mortality. Survival probabilities were estimated using the Kaplan-Meier method, and differences in the survival probabilities were analyzed using the log-rank test. Risk factors for waiting mortality were assessed using a Cox proportional hazards model forced entry. The proportional hazards assumption was checked using a complementary log-log plot. Missing data were not complemented. Differences were considered to be statistically significant when the *P*-value was less than 0.05. All analyses were performed using JMP® 18.0 (SAS Institute, Cary, NC, USA) and Prism 8.0 (GraphPad Software, San Diego, CA, USA) software.

## Results

### Patient characteristics

The characteristics of the 354 patients at the time of listing for LT from DBD are shown in Table 1. The median age was 48 years (range: 18–60). There were 168 female patients (47.5%) and 179 patients (49.4%) with a history of smoking. The median BMI and 6-minute walk distance values were 20.4 kg/m^2^ (range: 11.0–34.9) and 335 m (range: 0–753), respectively. The breakdown of disease categories was as follows: fibrotic pulmonary disease (n = 207 [58.5%]), pulmonary vascular disease (n = 65 [18.4%]), obstructive pulmonary disease (n = 38 [10.7%]), suppurative pulmonary disease (n = 22 [6.2%]) and allogeneic disease (n = 22 [6.2%]). Among the 354 patients, 147 (41.5%) survived to receive LT, and 102 (28.8%) were alive on the waiting list, while 105 (29.7%) died on the waiting list.

**Table 1:** Characteristics of the 354 Adult Patients at the Time of Registration for Lung Transplantation From Donation From Brain Death

Demographics		N (%) or median value (range)
Age at registration (years)		48 (18–60)
Sex	Female	168 (47.5%)
	Male	18 (52.5%)
Smoking history	Never	175 (49.4%)
	Former	179 (50.6%)
BMI (kg/m^2^)		20.4 (11.0–34.9)
6-minute walk distance (m)		335 (0–753)
Blood type	A	140 (39.6%)
	O	95 (26.8%)
	B	89 (25.1%)
	AB	30 (8.5%)
Disease category	Fibrotic	207 (58.5%)
	Vascular	65 (18.4%)
	Obstructive	38 (10.7%)
	Suppurative	22 (6.2%)
	Allogeneic	22 (6.2%)
Oxygen supplementation at rest*		193 (54.7%)
Mean PAP (mmHg)**		21 (7–97)
Lung function	FVC (L)***	2.0 (0.6–4.9)
	%FVC (%)***	54.2 (15.4–138.0)
	FEV1.0 (L)***	1.5 (0.3–3.7)
	FEV1.0/FVC (%)***	82.9 (18.8–100.0)
Laboratory data	pO_2_ (mmHg)****	74.2 (29.9–179.0)
	pCO_2_ (mmHg)****	40.7 (26.1–97.0)
	Albumin (g/dL)	4.0 (2.7–5.4)
	GNRI	98.37 (40.38–125.62)

BMI, body mass index; FEV1.0, forced expiratory volume in 1 s; FVC, forced vital capacity; GNRI, geriatric nutritional risk index; PAP, pulmonary arterial pressure; pCO_2_, partial pressure of carbon dioxide; pO_2_, partial pressure of oxygen.

*Data were missing for one patient.

**Data were missing for 21 patients.

***Data were missing for 10 patients.

****Data were missing for one patient.

### Cut-off value of the GNRI

The median GNRI value was 98.37 (range: 40.38–125.62), as shown in Table 1. A ROC curve for overall mortality revealed that the optimal cut-off value for the GNRI was 93.84, with a sensitivity, specificity, and area under the curve (AUC) of 71.9%, 49.5%, and 0.623, respectively (Supplementary Fig. 1).

### Association between the GNRI and patient characteristics

We divided the 354 patients into the low GNRI (<93.84; n = 122 [34.5%]) and high GNRI (≥ 93.84; n = 232 [65.5%]) groups based on the cut-off value of the GNRI. The low GNRI group had significantly more females (*P* = 0.007), a lower BMI (*P* < 0.001), more disease categories other than fibrotic pulmonary disease (*P* < 0.001), a shorter 6-minute walk distance (*P* = 0.002), oxygen supplementation at rest (*P* = 0.015), a lower forced vital capacity (FVC; *P* < 0.001), a lower forced expiratory volume in 1 s (FEV1.0; *P* < 0.001), a lower pO2 (*P* = 0.036), and a higher pCO2 (*P* < 0.001) compared with the high GNRI group (Table 2).

**Table 2:** Characteristics of the GNRI-low and GNRI-high Groups at the Time of Registration for Lung Transplantation from Donation After Brain Death

Demographics		Low GNRI group (< 93.84; n = 122 [34.5%])	High GNRI group (≥ 93.84; n = 232 [65.5%])	*P-*value
Age (years), median (range)		48 (18–60)	48 (18–60)	0.911
Sex	Female	70 (57.4%)	98 (42.2%)	0.007
	Male	52 (42.6%)	134 (57.8%)	
Smoking history	Never	72 (59.0%)	103 (44.4%)	0.009
	Former	50 (41.0%)	129 (55.6%)	
BMI (kg/m^2^), median (range)		17.0 (11.0–23.5)	22.4 (14.7–34.9)	< 0.001
Blood type	A	45 (36.9%)	95 (41.0%)	0.620
	O	38 (31.1%)	57 (24.6%)	
	B	29 (23.8%)	60 (25.8%)	
	AB	10 (8.28.5%)	20 (8.6%)	
Disease category	Fibrotic	59 (48.4%)	148 (63.8%)	< 0.001
	Vascular	18 (14.8%)	47 (20.3%)	
	Obstructive	17 (13.9%)	21 (9.1%)	
	Suppurative	16 (13.1%)	6 (2.6%)	
	Allogeneic	12 (9.8%)	10 (4.2%)	
6-min walk distance (m), median (range)		300 (10–591)	354 (0–753)	0.002
Oxygen supplementation at rest*	No	44 (36.4%)	116 (50.0%)	0.015
	Yes	77 (63.6%)	116 (50.0%)	
Mean PAP (mmHg), median (range)**		22 (7–92)	20 (7–97)	0.221
FVC (L), median (range)***		1.5 (0.7–4.6)	2.2 (0.6–4.9)	< 0.001
FEV1.0 (L), median (range)***		1.1 (0.3–3.6)	1.7 (0.4–3.7)	< 0.001
pO_2_ (mmHg), median (range)****		71.3 (29.9–171.5)	76.4 (41.0–179.0)	0.036
pCO_2_ (mmHg), median (range)****		44.9 (26.1–76.4)	39.2 (26.5–97.0)	< 0.001

BMI, body mass index; FEV1.0, forced expiratory volume in 1 s; FVC, forced vital capacity; PAP, pulmonary arterial pressure; SD, standard deviation.

*Data were missing for one patient.

**Data were missing for 21 patients.

***Data were missing for 10 patients.

****Data were missing for one patient.

### Analysis of the risk factors for waiting mortality

**Figure 1:**
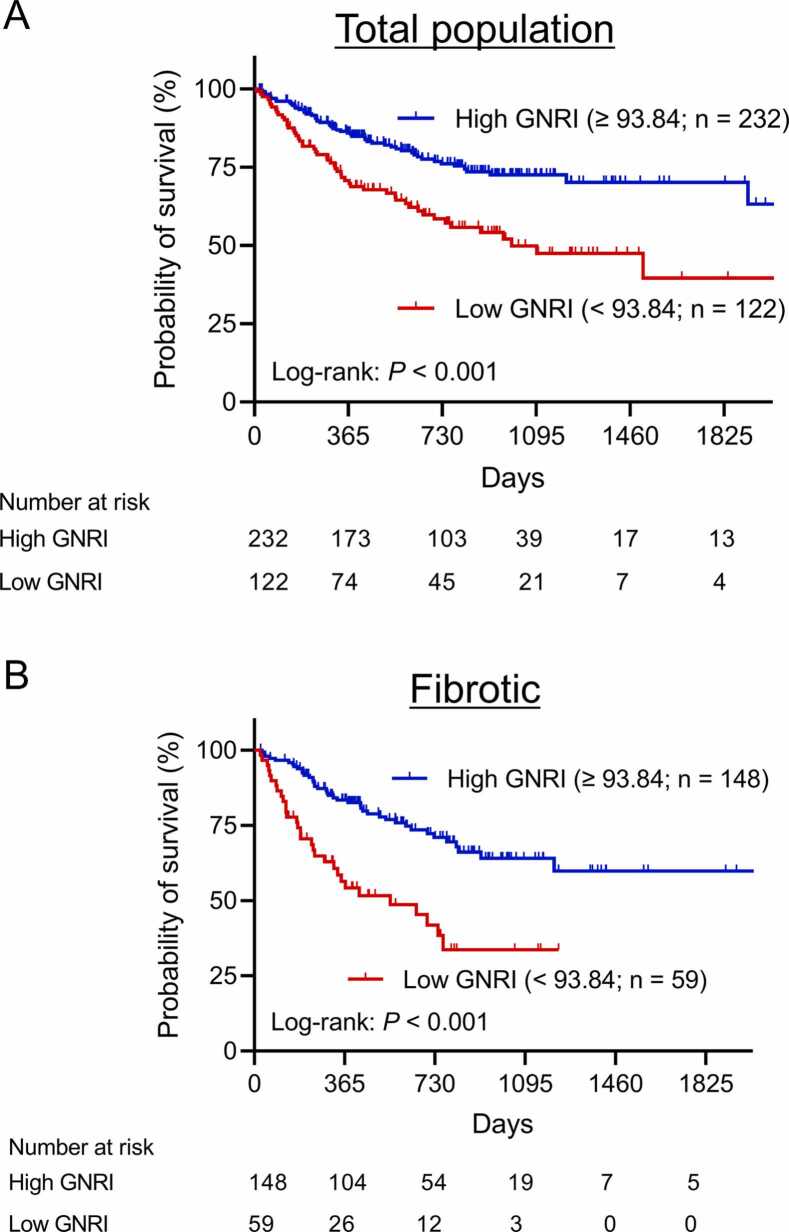
Overall survival of 354 adult patients registered for lung transplantation based on their GNRI. (A) Total population: low GNRI (< 93.84; n = 122) vs. high GNRI (≥ 93.84; n = 232). Log-rank: *P* < 0.001. (B) Fibrotic disease population: low GNRI (< 93.84; n = 59) vs. high GNRI (≥ 93.84; n = 148). Log-rank: *P* < 0.001. GNRI, geriatric nutritional risk index.

The median length of follow-up was 606 days. The low GNRI group had a significantly shorter median survival time than the high GNRI group (999 days versus not reached; *P* < 0.001; Fig. 1A). A subgroup analysis for each disease category showed that the patients with fibrotic pulmonary disease in the low GNRI group had a significantly shorter survival time than those in the high GNRI group (*P* < 0.001; Fig. 1B), while this association was not observed among patients with vascular, obstructive, suppurative and allogeneic diseases (Supplementary Figs. 2A-D). Multivariate analysis indicated that the significant independent prognostic factors for survival during the waiting period were the GNRI (hazard ratio [HR], 1.835; 95% confidence interval [CI], 1.085–3.105; *P* = 0.024), age (HR, 1.575; 95%CI, 1.006–2.466; *P* = 0.047), sex (HR, 0.545; 95%CI, 0.338–0.878; *P* = 0.013), 6-minute walk distance (HR, 2.321; 95%CI, 1.442–3.737; *P* < 0.001), forced vital capacity (HR, 1.889; 95%CI, 1.090–3.275; *P* = 0.023), and disease category (HR, 1.706; 95%CI, 1.037–2.808; *P* = 0.036; Table 3).

**Table 3:** Results of the Univariate and Multivariate Analyses of Mortality in Patients Registered for Cadaveric Lung Transplantation

Variables	Univariate Analysis			Multivariate Analysis		
	HR	95% CI	*P* value	HR	95%CI	*P* value
Age (≥ 47 years/<47 years)	2.125	1.410–3.201	< 0.001	1.575	1.006–2.466	0.047
Sex (female/male)	0.622	0.421–0.920	0.017	0.545	0.338–0.878	0.013
Smoking history (never/former)	0.679	0.460–1.001	0.051	0.805	0.506–1.282	0.362
BMI (< 17.4 kg/m^2^/≥ 17.4 kg/m^2^)	1.687	1.130–2.518	0.011	1.047	0.593–1.849	0.874
6-minute walk distance (< 313 m/≥ 313 m)	2.951	1.986–4.384	< 0.001	2.321	1.442–3.737	< 0.001
Oxygen supplementation at rest (yes/no)	1.480	0.998–2.195	0.051	1.040	0.648–1.671	0.870
FVC (< 1.3 L /≥ 1.3 L)	2.666	1.755–4.049	< 0.001	1.889	1.090–3.275	0.023
FEV1.0 (< 0.9 L /≥ 0.9 L)	1.693	1.102–2.600	0.016	1.225	0.719–2.085	0.456
Disease category (fibrotic/others)	1.974	1.306–2.983	0.001	1.706	1.037–2.808	0.036
GNRI (low [< 93.84]/high [≥ 93.84])	2.123	1.447–3.113	< 0.001	1.835	1.085–3.105	0.024

BMI, body mass index; CI, confidence interval; FEV1.0, forced expiratory volume in one second; FVC, forced vital capacity; GNRI, geriatric nutritional risk index; HR, hazard ratio.

### Validation of the prognostic impact of a GNRI of 93.84 in the validation cohort

The characteristics of the 80 patients in the validation cohort are shown in Supplementary Table 1. With the cut-off date set to July 2025, the median follow-up time for these patients was 413 days. Using a GNRI cut-off value of 93.84 derived from the study cohort, patients in the validation cohort were divided into those with high GNRI (n =54 [67.5%]) and low GNRI (n = 26 [32.5%]). Patients with a low GNRI exhibited a significantly shorter survival compared to those with high GNRI (*P* = 0.004; Supplementary Fig. 3).

### Perioperative significance of the GNRI in patients who underwent LT

We examined the association between the GNRI at the time of listing for LT and postoperative outcomes, including in-hospital death, length of ICU stay, length of mechanical ventilation, and length of in-hospital stay, among the 147 patients who underwent LT in the study cohort. Although there were no differences between the low GNRI group and the high GNRI group in in-hospital death (*P* = 0.527) and length of ICU stay (*P* = 0.140), the low GNRI tended to have a longer period for which mechanical ventilation was required (median length of mechanical ventilation: 8 days [1−455] vs. 5 days [1−368], *P* = 0.056) and a significantly longer length of hospital stay (median in-hospital stay: 56 days [17−456] vs. 42 days [24−409], *P* = 0.003) (Supplementary Table 2).

## Discussion

In the present study, the GNRI was an independent prognostic factor in patients awaiting LT from DBD. The GNRI is easily calculated using the serum albumin, patient body weight, and ideal body weight, and is useful in assessing the nutritional status of elderly patients and predicting their risk of in-hospital mortality.^5^ Furthermore, the GNRI has been reported to play a prognostic role in various diseases, including cancer, heart failure, and idiopathic pulmonary fibrosis.^6^, ^10^, ^11^ Importantly, the prognostic significance of the GNRI has also been reported in patients waiting for heart transplantation and in those who have undergone heart transplantation.^8^, ^9^ However, to the best of our knowledge, the present study is the first to clarify the significance of the GNRI in predicting the waitlist mortality of patients registered for LT from DBD. As the GNRI is calculated based on the patient’s body weight and serum albumin level, there is no requirement for any special diagnostic tests, suggesting that the GNRI can be easily applied in clinical settings.

The significance of the GNRI highlights the importance of body weight and serum albumin. Almost all studies of LT have used the BMI to assess the prognostic impact of body weight and have reported that a lower BMI results in poor survival among patients registered for LT and among those who have undergone LT.^12^, ^13^, ^14^ Furthermore, several studies have also demonstrated the prognostic role of the serum albumin in patients registered for LT and those who have undergone LT.^15^, ^16^, ^17^ In patients listed for LT, weight gain which does not reflect muscle mass can occur due to the use of steroids in patients with interstitial pneumonia or collagen tissue disease-associated interstitial lung disease. Additionally, patients with pulmonary arterial hypertension (either primary or secondary to the progressive lung diseases) may exhibit weight gain, suggesting that some patients may have a normal weight and BMI despite having a poor nutritional status. In the present study, among 232 patients with a BMI greater than 18.5 kg/m^2^, 22 (9.5%) exhibited low albumin levels of less than 3.5 g/dL (data not shown). Given these findings, monitoring both the body weight and serum albumin levels provides valuable insights into the overall health of patients, allowing clinicians to make more informed decisions regarding the timing of LT and potential preoperative nutritional interventions. In addition to this clinically important significance of the GNRI, the predictive ability of the current body weight / ideal body weight was improved by adding albumin (AUC of the weight ratio, albumin and GNRI: 0.568, 0.635 and 0.623, respectively).

Our findings have important implications for several clinical decisions. First, clinicians may need to more actively consider LT from marginal donors or living-donor LT for patients with a low GNRI. Lung allocation systems, such as CAS, are widely used to appropriately select patients for LT in Western countries. However, the GNRI may also serve as an allocation marker for LT, especially in countries like Japan, where there is a limited number of donors and a ‘first come first served’ principle for LT. Second, the GNRI could potentially serve as a referral criterion even in patients whose respiratory function does not meet the referral criteria. Third, nutritional intervention for patients with a low GNRI might reduce the waitlist mortality, although it is not yet clear which types of nutritional support are effective. Fourth, the significant association between the GNRI at the time of listing and the length of in-hospital stay in 147 patients who underwent LT suggests that patients with a low GNRI may require more time to recover after LT than those with a high GNRI. Future studies evaluating the effectiveness of various types of nutritional support should be conducted to reduce the waitlist mortality of patients registered for LT and improve postoperative outcomes.

A previous study defined the cut-off value of the GNRI as 98 based on the cut-off values for the serum albumin and weight loss in elderly patients.^5^ Patients with a GNRI of less than 98 are reportedly significantly associated with an increased risk of mortality and complications compared with those with a GNRI of 98 or above. Some subsequent studies also adopted 98 as the cut-off value for the GNRI, while others used cut-off values of 82, 92, and 94.^6^, ^8^, ^9^, ^10^, ^11^ Although our study determined that the optimal cut-off value of the GNRI was 93.84 based on a ROC curve for waitlist mortality, the survival curves were significantly different between patients with a GNRI of less than 98 and those with a GNRI of 98 or above (Supplementary Fig. 4). However, we think that the GNRI cut-off value should differ between studies because there are differences between studies in the patient background, including age and disease. Future studies with a larger number of patients registered for LT from DBD are warranted to validate the present results.

The present study has several limitations. First, its retrospective and single-center nature may have resulted in selection bias. Second, although the total number of patients was large, the sample size in each disease category was small, especially for disease categories other than fibrotic pulmonary disease, potentially leading to inappropriate statistical analyses. Third, the inclusion of only Japanese patients may limit the generalizability of the findings, as there are differences between countries in the waiting period prior to LT and the disease, physical, and nutritional characteristics of patients. Fourth, as patients who received LT were censored at the time of transplantation, this could introduce informative censoring, potentially biasing the survival analysis. Fifth, although our findings obtained from the study cohort were validated using the validation cohort at our institution, the sample size was small and the follow-up period was short. Lastly, the serum albumin level is highly variable, and episodes of critical illness can lead to decreases in albumin, indicating the problem with timing of the serum albumin measurement. There is the potential for significant inherent bias in the score if subjects who are acutely ill undergo urgent assessment and have low albumin scores but are compared to subjects evaluated and listed in a period of stability who have higher albumin scores. This problem is exacerbated in a population that has a wide range of wait list times. Furthermore, in our study cohort, the median period between albumin measurement and listing for LT was 102 days (range: 11–660 days), which suggests the potential for changes in albumin levels during this period. The appropriate timing of albumin measurement, including trajectory changes, during the waiting period for LT should be investigated in future studies.

In conclusion, a greater risk of malnutrition at the time of LT listing, as indicated by the GNRI, was independently associated with a higher risk of mortality during the waiting period. Therefore, the GNRI might represent a promising new prognostic marker when selecting candidates for LT (especially those with fibrotic disease) and identifying those who may benefit from nutritional interventions prior to LT.

## Ethical statement and consent for publication

This retrospective, descriptive, exploratory study of an ongoing cohort was approved by the Ethics Committee of The University of Tokyo Hospital [IRB#: 2406-(8), April 12th, 2023]. Due to its retrospective nature, the description of this study was provided to all patients through an opt-out process, giving them the option to decline the use of their information.

## Funding

The authors declare there were no funding sources for this study.

## CRediT authorship contribution statement

All authors meet the ICMJE authorship criteria. Each author’s contributions were as follows: (I) Conception and design: GT; (II) Administrative support: MS; (III) Provision of study materials or patients: GT, MY, TY, TA, CK and MS; (IV) Collection and assembly of data: GT, MY, TY, and CK; (V) Data analysis and interpretation: GT, MK and MS; (VI) Manuscript writing: all authors; (VII) Final approval of manuscript: all authors.

All authors have approved the submitted version of the manuscript and agreed to be accountable for any part of the work.

## Declaration of Competing Interest

The authors declare that they have no known competing financial interests or personal relationships that could have appeared to influence the work reported in this paper. We have nothing to disclose.

## Appendix A Supplemental material

.

Supplemental Figure 1. Receiver operating characteristic curve for the GNRI in predicting waitlist mortality. The area under the curve is shown to assess the sensitivity and specificity of the GNRI with a cut-off value of 93.84. GNRI, geriatric nutritional risk index

.

Supplemental Figure 2. Overall survival of patients with vascular and obstructive diseases based on their GNRI. (A) Vascular disease: low GNRI (< 93.84; n = 18) vs. high GNRI (≥ 93.84; n = 47). Log-rank: *P* = 0.128. (B) Obstructive disease: low GNRI (< 93.84; n = 17) vs. high GNRI (≥ 93.84; n = 21). Log-rank: *P* = 0.503. (C) Suppurative disease: low GNRI (< 93.84; n = 16) vs. high GNRI (≥ 93.84; n = 6). Log-rank: *P* = 0.105. (D) Allogeneic disease: low GNRI (< 93.84; n = 12) vs. high GNRI (≥ 93.84; n = 10). Log-rank: *P* = 0.882. GNRI, geriatric nutritional risk index

.

Supplemental Figure 3. Overall survival of 80 patients in the validation cohort using the GNRI cut-off value of 93.84. Low GNRI (< 93.84; n = 26) vs. high GNRI (≥ 93.84; n = 54). Log-rank: *P* = 0.004. GNRI, geriatric nutritional risk index

.

Supplemental Figure 4. Overall survival of patients registered for lung transplantation using the GNRI cut-off value of 98. Low GNRI (< 98; n = 172) vs. high GNRI (≥ 98; n = 182). Log-rank: *P* = 0.007. GNRI, geriatric nutritional risk index

## Footnotes

Appendix A Supplemental data associated with this article can be found in the online version at doi:10.1016/j.jhlto.2025.100424.
