# Supplementary material for: Prognostic impact of the geriatric nutritional risk index on waitlist mortality in adult patients listed for lung transplantation from donation after brain death
Source: JHLT Open. 2025 Dec 5;11:100424. doi: 10.1016/j.jhlto.2025.100424 (PMC12800619; doi:10.1016/j.jhlto.2025.100424)
Supplement: Supplementary file 1 — Supplemental material [file mmc1.docx]

**Supplementary Table 1.** Characteristics of the 80 patients in the validation cohort at the time of registration for lung transplantation.

| **Demographics** |  | **N (%) or median value (range)** |
| --- | --- | --- |
| Age at registration (years) |  | 50 (18–59) |
| Sex | Female | 39 (48.8%) |
|  | Male | 41 (51.2%) |
| Blood type | A | 27 (33.8%) |
|  | O | 26 (32.5%) |
|  | B | 23 (28.7%) |
|  | AB | 4 (5.0%) |
| Disease category | Fibrotic | 55 (68.8%) |
|  | Vascular | 8 (10.0%) |
|  | Obstructive | 7 (8.7%) |
|  | Suppurative | 3 (3.8%) |
|  | Allogeneic | 7 (8.7%) |
| GNRI |  | 102.146 (78.10–125.81) |

GNRI, geriatric nutritional risk index.

**Supplementary Table 2.** Characteristics and postoperative outcomes of the 147 patients who underwent lung transplantation.

| **Demographics** |  | **Low GNRI group**  **(< 93.84; n = 47 [32.0%])** | **High GNRI group**  **(****≥ 93.84; n = 100 [68.0%])** | ***P-*value** |
| --- | --- | --- | --- | --- |
| Age at lung transplantation (years), median (range) | | 50 (20–62) | 48 (20–63) | 0.907 |
| Sex | Female | 26 (55.3%) | 40 (40.0%) | 0.082 |
|  | Male | 21 (44.7%) | 60 (60.0%) |  |
| Blood type | A | 13 (27.7%) | 40 (40.0%) | 0.482 |
|  | O | 16 (34.0%) | 29 (29.0%) |  |
|  | B | 12 (25.5%) | 23 (23.0%) |  |
|  | AB | 6 (12.8%) | 8 (8.0%) |  |
| Disease category | Fibrotic | 20 (42.5%) | 63 (63.0%) | < 0.001 |
|  | Vascular | 5 (10.6%) | 21 (21.0%) |  |
|  | Obstructive | 6 (12.8%) | 10 (10.0%) |  |
|  | Suppurative | 10 (21.3%) | 3 (3.0%) |  |
|  | Allogeneic | 6 (12.8%) | 3 (3.0%) |  |
| In-hospital death | No | 44 (93.6%) | 96 (96.0%) | 0.527 |
|  | Yes | 3 (5.7%) | 4 (4.0%) |  |
| Length of ICU stay (days), median (range) | | 15 (3–284) | 12 (4–371) | 0.140 |
| Length of mechanical ventilation, median (range) | | 8 (1–455) | 5 (1–368) | 0.056 |
| Length of in-hospital stay, median (range) | | 56 (17–456) | 42 (24–409) | 0.003 |

GNRI, geriatric nutritional risk index; ICU: intensive care unit.
