# Supplementary figures and images for: Prognostic impact of the geriatric nutritional risk index on waitlist mortality in adult patients listed for lung transplantation from donation after brain death
Source: JHLT Open. 2025 Dec 5;11:100424. doi: 10.1016/j.jhlto.2025.100424 (PMC12800619; doi:10.1016/j.jhlto.2025.100424)

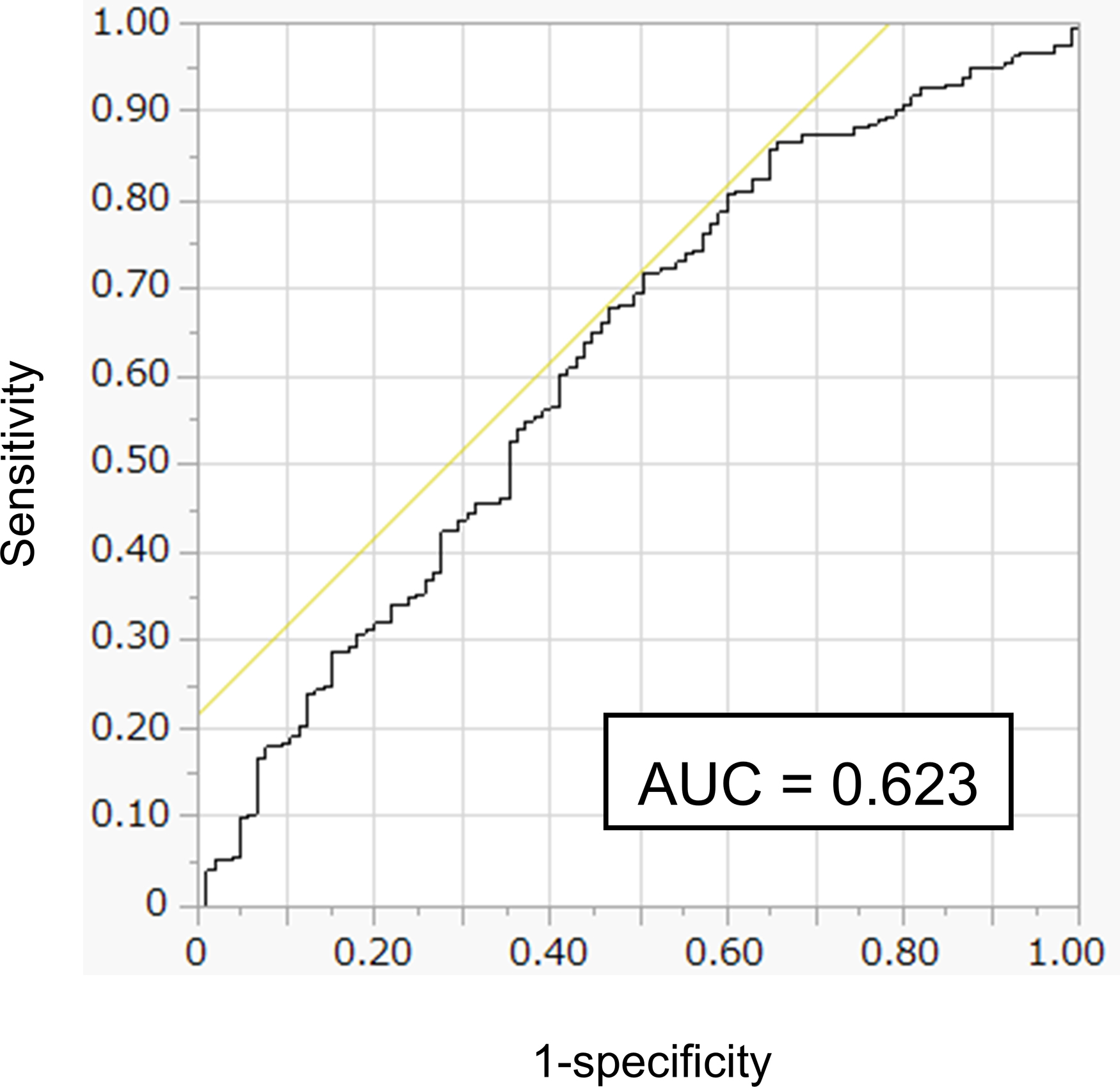

Supplement: Supplementary file 2 — Supplemental Figure 1. Receiver operating characteristic curve for the GNRI in predicting waitlist mortality. The area under the curve is shown to assess the sensitivity and specificity of the GNRI with a cut-off value of 93.84. GNRI, geriatric nutritional risk index [file mmc2.jpg]

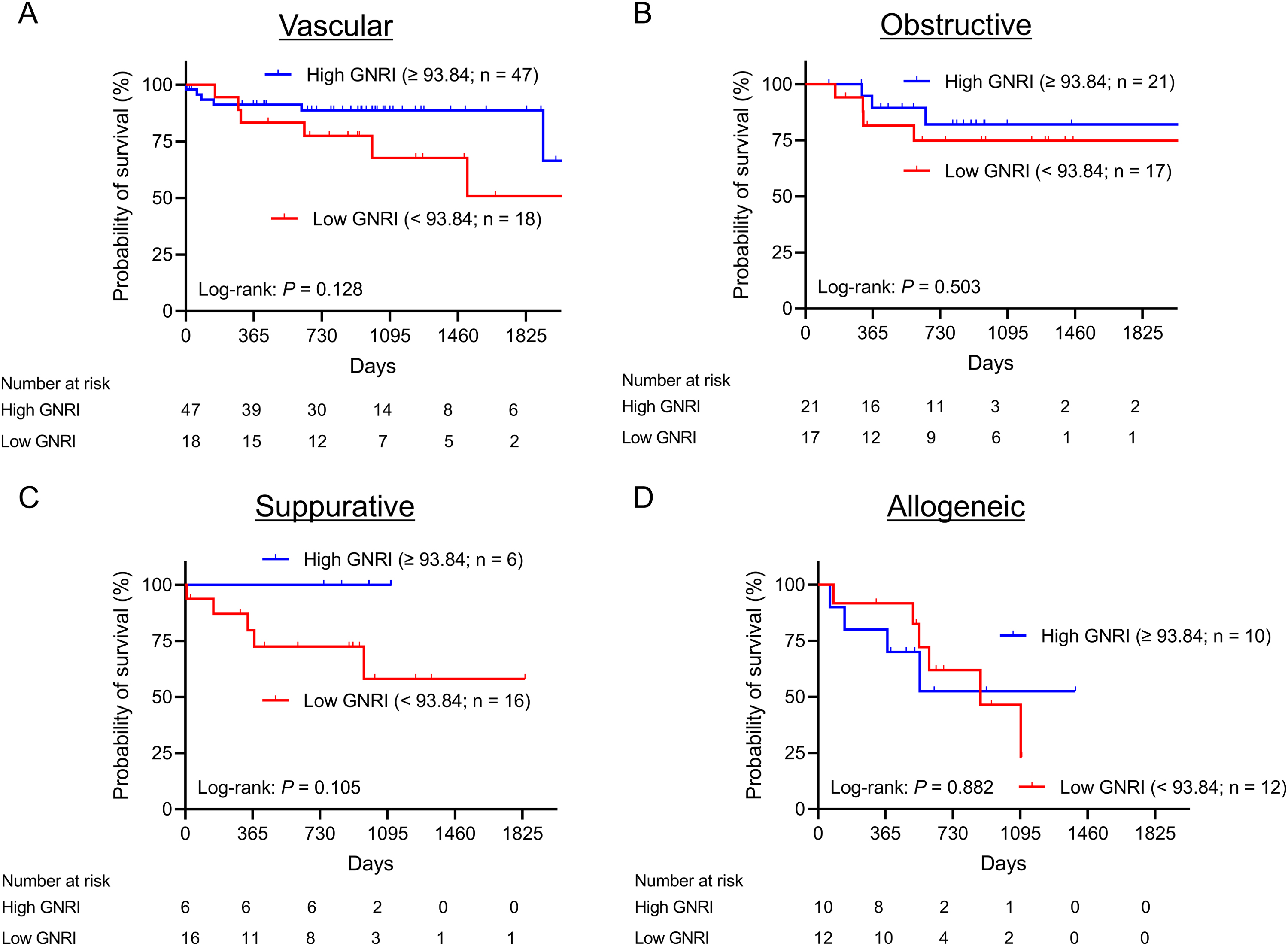

Supplement: Supplementary file 3 — Supplemental Figure 2. Overall survival of patients with vascular and obstructive diseases based on their GNRI. (A) Vascular disease: low GNRI (< 93.84; n = 18) vs. high GNRI (≥ 93.84; n = 47). Log-rank: P = 0.128. (B) Obstructive disease: low GNRI (< 93.84; n = 17) vs. high GNRI (≥ 93.84; n = 21). Log-rank: P = 0.503. (C) Suppurative disease: low GNRI (< 93.84; n = 16) vs. high GNRI (≥ 93.84; n = 6). Log-rank: P = 0.105. (D) Allogeneic disease: low GNRI (< 93.84; n = 12) vs. high GNRI (≥ 93.84; n = 10). Log-rank: P = 0.882. GNRI, geriatric nutritional risk index [file mmc3.jpg]

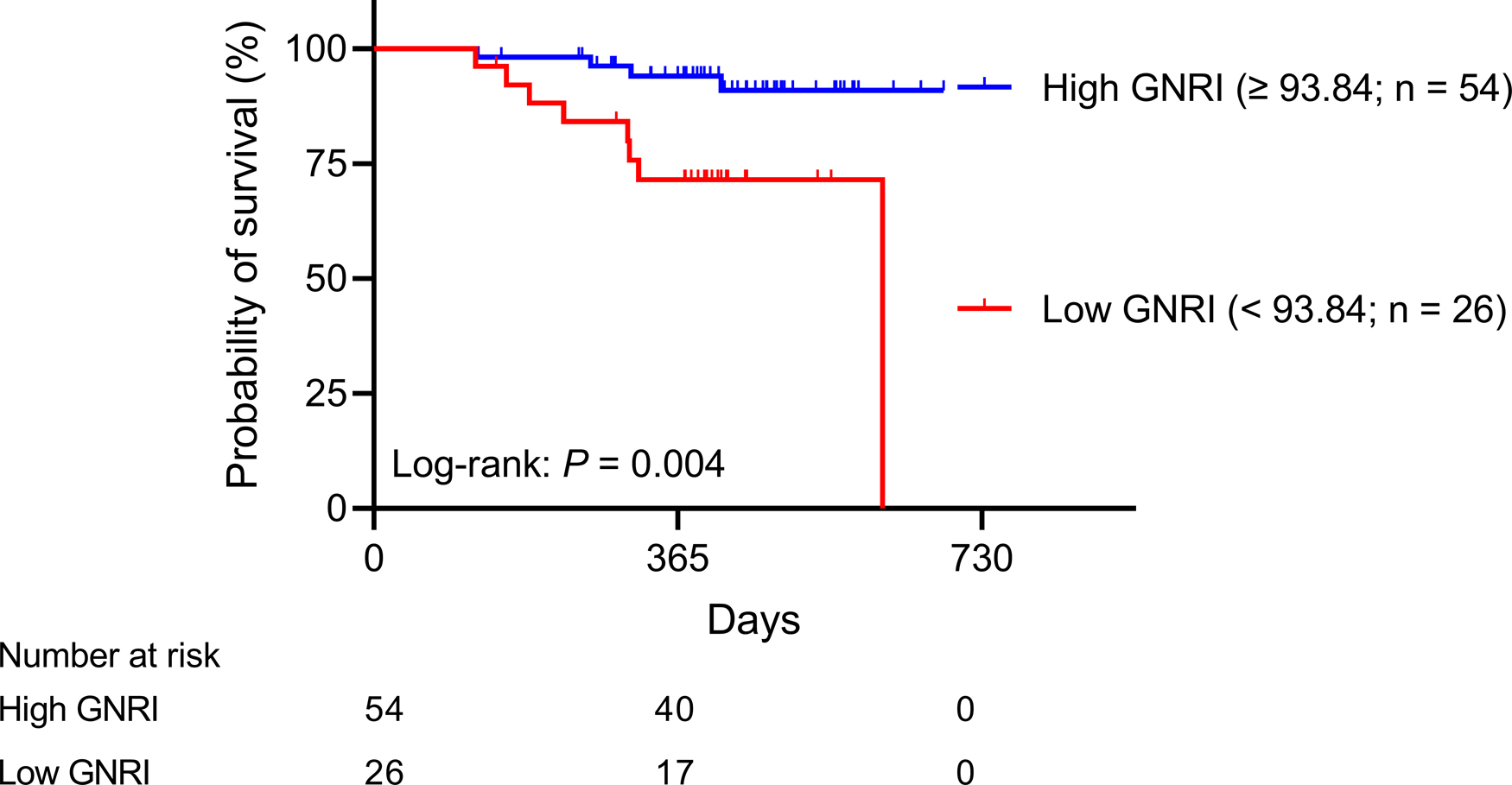

Supplement: Supplementary file 4 — Supplemental Figure 3. Overall survival of 80 patients in the validation cohort using the GNRI cut-off value of 93.84. Low GNRI (< 93.84; n = 26) vs. high GNRI (≥ 93.84; n = 54). Log-rank: P = 0.004. GNRI, geriatric nutritional risk index [file mmc4.jpg]

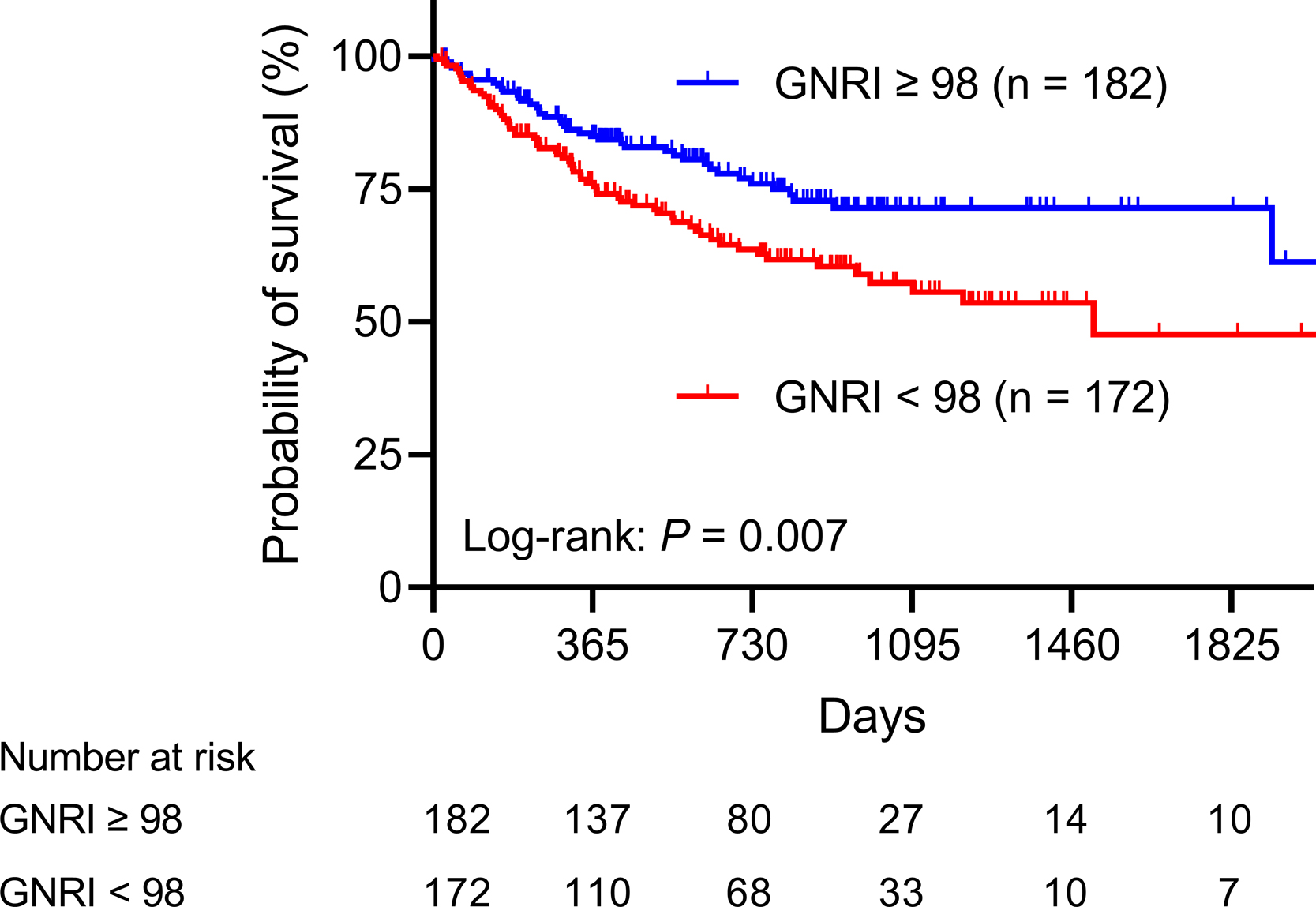

Supplement: Supplementary file 5 — Supplemental Figure 4. Overall survival of patients registered for lung transplantation using the GNRI cut-off value of 98. Low GNRI (< 98; n = 172) vs. high GNRI (≥ 98; n = 182). Log-rank: P = 0.007. GNRI, geriatric nutritional risk index [file mmc5.jpg]
